# Circulating Mucosal Associated Invariant T Cells Are Activated in *Vibrio cholerae* O1 Infection and Associated with Lipopolysaccharide Antibody Responses

**DOI:** 10.1371/journal.pntd.0003076

**Published:** 2014-08-21

**Authors:** Daniel T. Leung, Taufiqur R. Bhuiyan, Naoshin S. Nishat, Mohammad Rubel Hoq, Amena Aktar, M. Arifur Rahman, Taher Uddin, Ashraful I. Khan, Fahima Chowdhury, Richelle C. Charles, Jason B. Harris, Stephen B. Calderwood, Firdausi Qadri, Edward T. Ryan

**Affiliations:** 1 Centre for Vaccine Sciences, International Centre for Diarrhoeal Disease Research, Bangladesh (icddr,b), Dhaka, Bangladesh; 2 Division of Infectious Disease, Massachusetts General Hospital, Boston, Massachusetts, United States of America; 3 Department of Medicine, Harvard Medical School, Boston, Massachusetts, United States of America; 4 Department of Pediatrics, Harvard Medical School, Boston, Massachusetts, United States of America; 5 Department of Microbiology and Immunobiology, Harvard Medical School, Boston, Massachusetts, United States of America; 6 Department of Immunology and Infectious Diseases, Harvard School of Public Health, Boston, Massachusetts, United States of America; University of California San Diego School of Medicine, United States of America

## Abstract

**Background:**

Mucosal Associated Invariant T (MAIT) cells are innate-like T cells found in abundance in the intestinal mucosa, and are thought to play a role in bridging the innate-adaptive interface.

**Methods:**

We measured MAIT cell frequencies and antibody responses in blood from patients presenting with culture-confirmed severe cholera to a hospital in Dhaka, Bangladesh at days 2, 7, 30, and 90 of illness.

**Results:**

We found that MAIT (CD3^+^CD4^−^CD161^hi^Vα7.2^+^) cells were maximally activated at day 7 after onset of cholera. In adult patients, MAIT frequencies did not change over time, whereas in child patients, MAITs were significantly decreased at day 7, and this decrease persisted to day 90. Fold changes in MAIT frequency correlated with increases in LPS IgA and IgG, but not LPS IgM nor antibody responses to cholera toxin B subunit.

**Conclusions:**

In the acute phase of cholera, MAIT cells are activated, depleted from the periphery, and as part of the innate response against *V. cholerae* infection, are possibly involved in mechanisms underlying class switching of antibody responses to T cell-independent antigens.

## Author Summary

*Vibrio cholerae* is the bacterium that causes cholera, which can be a potentially fatal diarrheal disease that affects millions of people worldwide each year. How our immune system provides protection against cholera is poorly understood. Mucosal Associated Invariant T (MAIT) cells are recently discovered immune cells found in the blood and intestinal tract of humans. In this study of cholera patients in Dhaka, Bangladesh, we found that blood MAIT cells are activated during cholera, and that in children, blood MAIT cells are decreased in number during the course of disease. We also found that the MAIT cell response correlates with the antibody response to *V. cholerae* O1 lipopolysaccharide, which in the past has been shown to be an important determinant of protection. These findings suggest that MAIT cells may play an important role in the body's defense against cholera.

## Introduction

Cholera is an acutely dehydrating diarrheal disease caused predominantly by *Vibrio cholerae* O1 infection [1]. It is endemic in 50 countries, causing up to 3 million cases and 100,000 deaths annually [2]. In humans, ingestion of contaminated water or food leads to colonization by the pathogen of the small intestine and the subsequent toxin-mediated secretion of fluid can result in rapid dehydration and death due to hypovolemic shock [1].

The mechanisms of protection against cholera are not well understood. Immunity to cholera is serogroup specific, with serogroup differentiated by the O-specific polysaccharide (OSP) of the lipopolysaccharide (LPS) of *V. cholerae* [3]. In studies of household contacts of cholera patients, we have shown that LPS-specific antibody and memory B cell responses to LPS are associated with protection from disease [4], [5]. We have demonstrated, in patients hospitalized with acute severe cholera, that *V. cholerae* O1 infection induces significant increases in circulating antigen-specific antibody, antibody secreting cell, and memory B cell responses, as well as antigen-specific memory T cell responses in both children and adults [6]–[10]. Using duodenal biopsies in adults and rectal biopsies in children, we have shown that effectors of the innate response are upregulated during cholera [11]–[14]. However, the relationship between the innate and adaptive immune responses to cholera remains poorly defined.

Mucosal Associated Invariant T (MAIT) cells are recently described innate-like T cells. Originally defined by an invariant T cell receptor α segment (Vα7.2) and high expression of CD161, they have an effector memory phenotype and tissue-homing surface markers [15]. MAIT cells represent up to 10% of circulating T cells in healthy adults from high-income countries, and are found in abundance in the intestinal mucosa, mesenteric lymph nodes, and the liver [16]. They are MHC-related 1 (MR1) restricted, and are activated by vitamin B metabolites of various bacterial and fungal species [17]. In acute pulmonary bacterial and mycobacterial infections in humans, MAIT cells are enriched at mucosal sites and are depleted in the periphery [18], [19]. In human HIV infection, MAIT cells are chronically depleted in the periphery, but numbers are maintained in the gut mucosa, though at all sites they are activated but functionally exhausted [20], [21]. MAIT cells have been associated with protection from a number of bacterial infections in animal models [18], [22]–[24], and recent studies have implicated their association with clinical outcomes in bacterial sepsis [25] and antibody secreting cell responses to an oral *Shigella* vaccine [26]. However, the kinetics of MAIT cell responses following human mucosal infection have yet to be defined, and their role in bridging innate and adaptive immune responses is not well understood.

We were therefore interested in assessing MAIT cell levels during human *V. cholerae* O1 infection, and to explore the relationship of MAIT cells with adaptive B cell responses during cholera.

## Methods

### Study population

We enrolled patients hospitalized at the Dhaka Hospital of the icddr,b who presented with severe acute watery diarrhea and stool culture positive for *V. cholerae* O1. We excluded patients under 2 years of age and those who were co-infected with other enteric pathogens by conventional stool culture techniques or had parasites identified by microscopic examination of stool. We defined children as those aged 2–17 years, and adults as aged ≥18 years. After informed consent of patients or parents/guardians, we obtained blood by venipuncture on days 2, 7, 30, and 90 following presentation. We recruited age-matched healthy controls from an informal settlement area with a similar socioeconomic status as that of areas where hospitalized patients resided. We excluded controls who had any diarrhea in the previous 2 weeks or any fever or antibiotic use in the previous week.

### Phenotyping of MAIT cells

**Figure 1:**
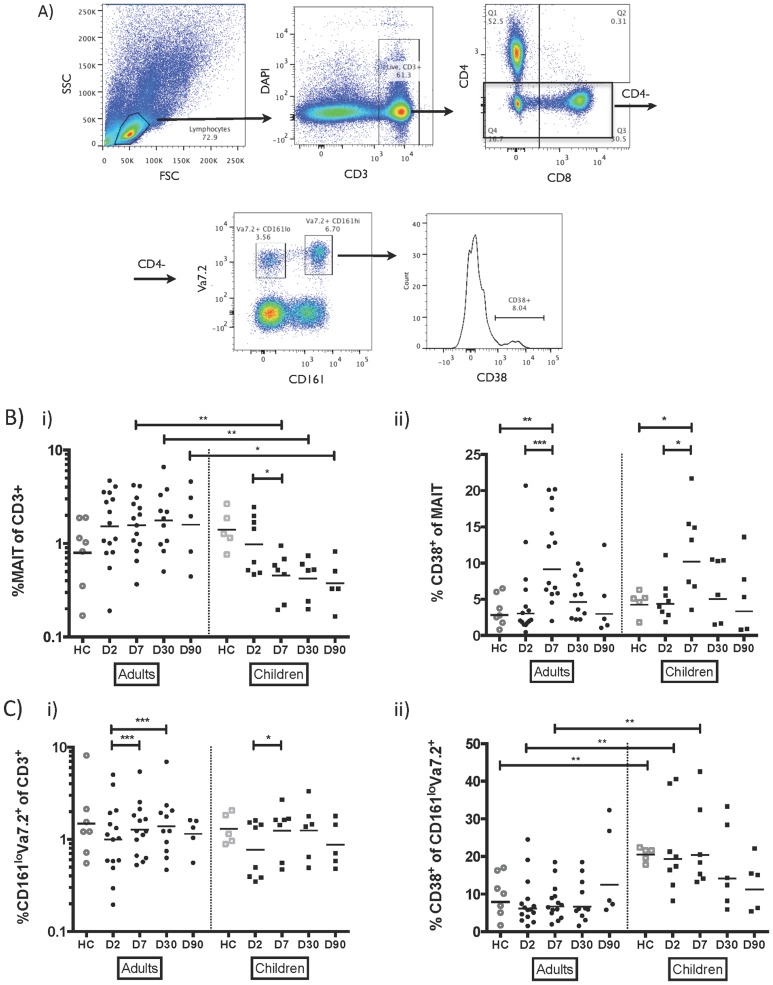
MAIT cells are activated in cholera patients, and depleted in children with cholera. A) Gating strategy for phenotyping of MAIT cells. Geometric mean of frequencies of B) MAIT and C) CD4^−^CD161^lo^Va7.2^+^ cells of healthy controls and patients with severe cholera, separated by adults and children, as i) proportion of CD3^+^ cells, and ii) activated (CD38^+^) cells as proportion of parent. * P<0.05; ** P<0.01; *** P<0.001.

We isolated peripheral blood mononuclear cells (PBMCs) and plasma from heparinized blood by density gradient centrifugation on Ficoll-Isopaque (Pharmacia, Piscataway, NJ). We stored plasma at −20°C for further immunological analysis. We immediately washed and stained the freshly isolated PBMCs with the following fluorochrome-conjugated antibodies purchased from BioLegend, BD, or Invitrogen: Vα7.2-PE, CD3-PE-Texas Red, CD4-Amcyan, CD8-FITC, CD161-APC, CD38-PE-Cy7, and DAPI. After 45 minutes incubation at 4°C, we analyzed at least 10^5^ lymphocytes on a FACSAria III flow cytometer (BD Biosciences, San Jose, CA) and analyzed data using FlowJo 10 software (TreeStar Inc, Ashland, OR). We used Cytometer Setup & Tracking beads (BD Biosciences) to check for inter-day variability, and Fluorescence Minus One (FMO) controls. The gating strategy is shown in Figure 1A. We defined MAIT cells as live (DAPI^−^) CD3^+^CD4^−^CD161^hi^Vα7.2^+^ cells, expressed as a percentage of total CD3^+^ lymphocytes, and used CD38 as a marker of cell activation. We also assessed the frequency of circulating live CD3^+^CD4^−^CD161^lo^Vα7.2^+^ cells, which have been suggested to be MAIT-derived cells associated with absent or reduced cytokine secretion *in vitro* [18], [20], [26], [27], and also associated with MAIT cell loss and functional exhaustion in HIV patients [20].

### Plasma antibody levels

We measured plasma cholera toxin B subunit (CtxB; gift from Ann-Mari Svennerholm, University of Gothenburg) and LPS (prepared from *V. cholerae* O1 as previously described [28])-specific IgA, IgG, and IgM antibody responses of patients using a standardized ELISA technique as previously described [29].

### Statistical analysis

We assessed differences in immune responses between days after presentation of patients by Wilcoxon signed rank test, and between healthy controls and patients by Mann-Whitney U test. To determine the association between MAIT and plasma antibody responses, we used Spearman's correlation. All P values were two-tailed, with a value of <0.05 considered the threshold for statistical significance. We performed analyses using SPSS version 17.0 (SPSS Inc., Chicago, IL), and GraphPad Prism version 6.0 (GraphPad Software, Inc., La Jolla, CA).

### Ethics statement

This study was approved by the Ethical Review and Research Review Committees of the icddr,b and the Institutional Review Board of Massachusetts General Hospital. Written informed consent was obtained from guardians of child participants (<18 years), and adult participants (≥18 years) provided their own consent.

## Results

We enrolled a total of 23 cholera patients (15 adults and 8 children) between March and June 2013, with 21, 17, and 10 patients completing day 7, 30, and 90 follow-up, respectively (Table 1). We enrolled 7 adult and 5 child healthy controls, which were not significantly different in age or blood group than patients. While a higher percentage of healthy controls than patients were female, we did not see any differences in MAIT frequency between genders (data not shown).

**Table 1:** Enrollment, follow-up, and demographics of study participants.

	Adults		Children	
	Patients	Healthy Controls	Patients	Healthy Controls
Enrolled	15	7	8	5
Completed 7 day f/u	14	n/a	7	n/a
Completed 30 day f/u	11	n/a	6	n/a
Completed 90 day f/u	5	n/a	5	n/a
Age, median (range)	32 (20–45)	30 (21–42)	5.5 (3–11.5)	6 (3–9)
Sex, # females (%)	1 (7) *	5 (71) *	1 (13)	3 (60)

f/u = follow-up;

* P<0.05 by Fisher exact test.

### MAIT cells are activated in cholera and frequencies are decreased in children

We show the kinetics of circulating MAIT cell frequencies after acute *V. cholerae* infection in Figure 1B. In adults, the frequencies of MAIT cells in peripheral blood of cholera patients were not different than those of healthy controls, and no changes were seen for up to 90 days. Conversely, in child patients, the frequency of MAIT cells decreased from day 2 to day 7 of presentation (P<0.05). This decreased frequency persisted up to day 90, though the change was not statistically significant after day 7, in part due to smaller numbers of children following up at later days. There were no differences in CD8 expression within MAITs during the course of infection in either children or adults. In both children and adults, the proportion of MAIT cells in peripheral blood with the activation marker CD38 were significantly increased at day 7 compared to day 2 following presentation and healthy controls (P<0.001 for adults and P<0.05 for children, Figure 1B ii), and returned to baseline levels by day 30. Similar findings were seen when frequencies were analyzed as absolute count per mm^3^ of blood (Figure S1).

### CD38^+^CD161^lo^Vα7.2^+^ cells are inversely correlated with MAIT responses

**Figure 2:**
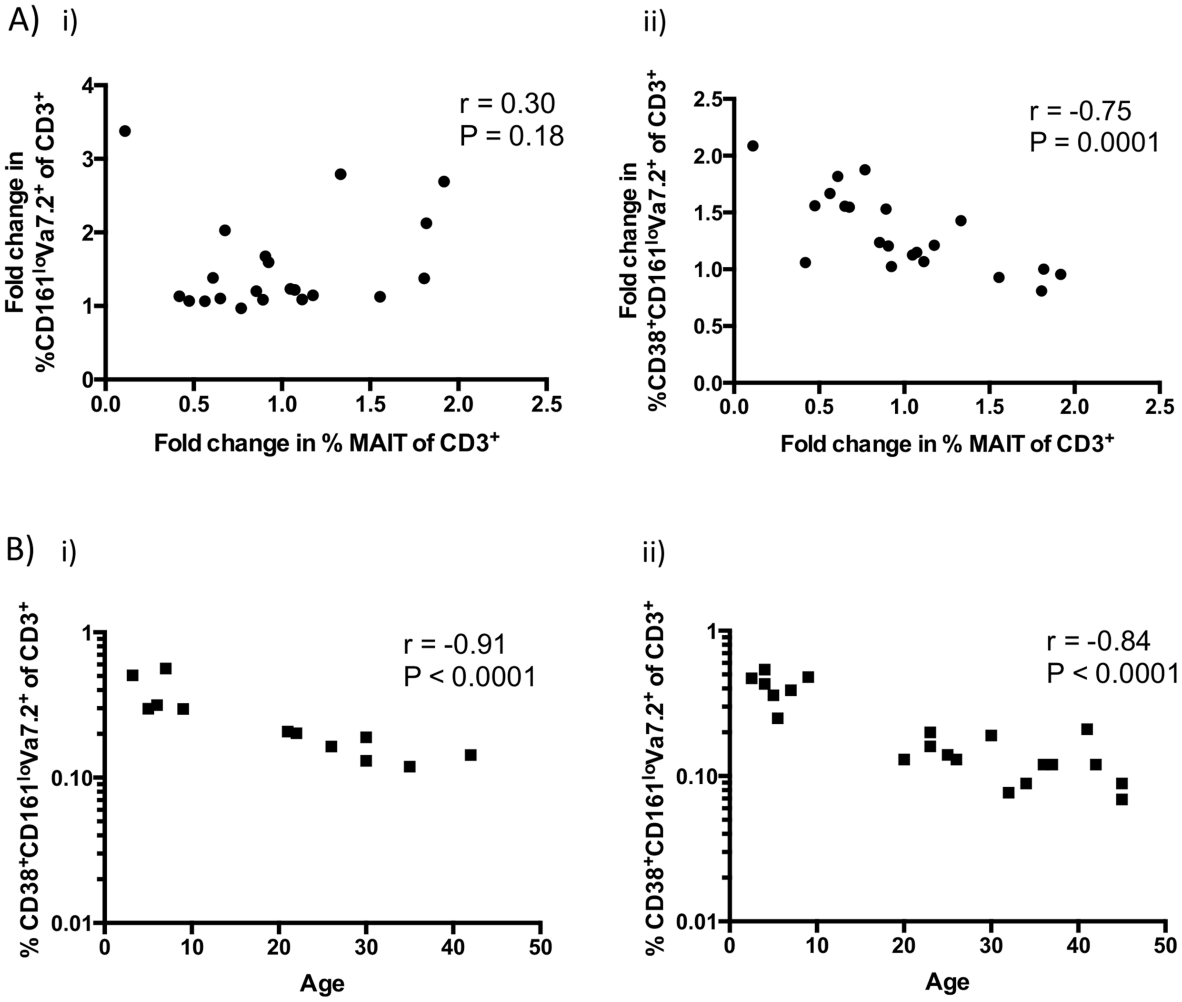
CD38^+^CD161^lo^Vα7.2^+^ cells are inversely associated with age and changes in MAIT cells. A) Spearman correlation of day 2 to day 7 fold changes in %MAIT cells with fold changes across the same days in i) CD161^lo^Vα7.2^+^, and ii) CD38^+^CD161^lo^Vα7.2^+^ cells. B) Spearman correlation of CD38^+^CD161^lo^Vα7.2^+^ cells with age for i) healthy controls and ii) cholera patients on day 7 after presentation.

The frequency of CD161^lo^Vα7.2^+^ cells as a proportion of CD3^+^ cells increased from day 2 to day 7 following presentation with cholera in both children (P<0.05) and adults (P<0.001), and in adults, this increase was statistically significant up to day 30 compared to baseline (Figure 1C i). While we did not see any changes in percent of CD161^lo^Vα7.2^+^ cells expressing the activation marker CD38^+^ between time points, we saw a significantly higher proportion of activated CD161^lo^Vα7.2^+^ cells in children than adults in both healthy controls and at days 2 and 7 after presentation (P<0.01, Figure 1C ii). Based on data suggesting that CD161^lo^Vα7.2^+^ cells may be derived from co-culture of MAIT cells with bacteria, and that MAIT cell loss is associated with an increase in CD161^lo^Vα7.2^+^ cells in HIV patients [20], we assessed the relationship between MAIT cell loss and CD161^lo^Vα7.2^+^ cells. We found that fold changes in MAIT cell frequency at day 7 compared to baseline (day 2 after presentation) were not correlated with fold changes in CD161^lo^Vα7.2^+^ cell frequency (r = 0.30, P = 0.18, Figure 2A i), but were inversely correlated with fold changes in activated (CD38^+^) CD161^lo^Vα7.2^+^ T cells (r = −0.75, P = 0.0001, Figure 2A ii). When expressed as percentage of CD3^+^ cells, CD38^+^CD161^lo^Vα7.2^+^ cells were negatively correlated with age in both healthy controls (r = −0.91, P<0.0001, Figure 2B i) and patients (P<0.01 for all days; representative day 7 shown in Figure 2B ii).

### Fold changes in MAIT cells are correlated with fold changes in class-switched LPS antibody responses

**Figure 3:**
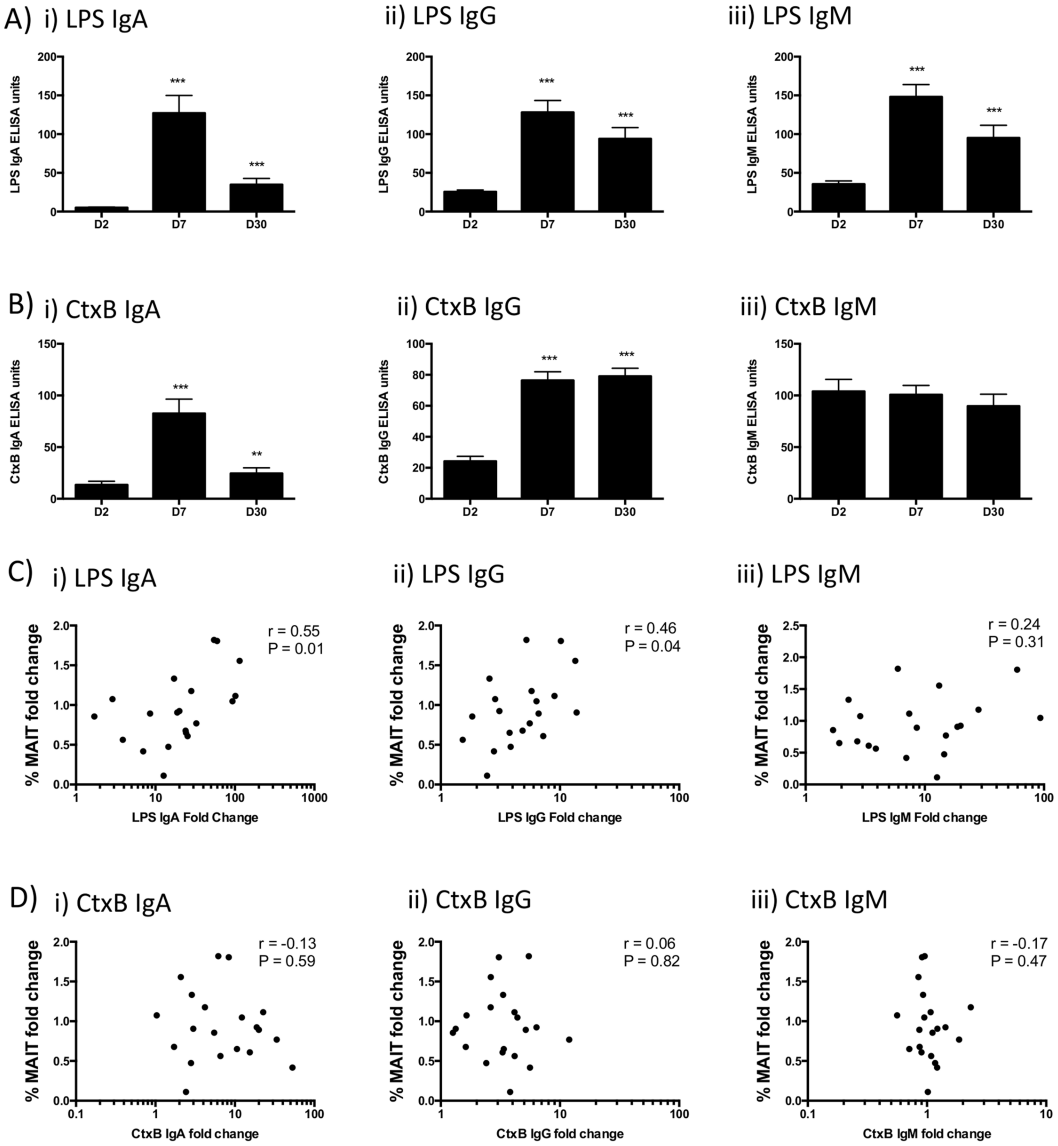
Class-switched antibody responses against LPS are correlated with MAIT cell responses. Antibody responses against A) LPS, and B) CtxB, of cholera patients at days 2, 7, and 30 after hospitalization, displayed as mean with standard errors. Compared with day 2, * P<0.05; ** P<0.01; *** P<0.001. Correlation of fold changes in %MAIT cells and fold changes in antibody response against C) LPS, and D) CtxB, of cholera patients.

We determined the plasma antibody responses in patients to two *V. cholerae* antigens, LPS (a T cell-independent antigen) and CtxB (a T cell-dependent antigen) (Figure 3A, B). We showed that the day 2 to day 7 fold change of frequency of MAITs as a proportion of CD3^+^ cells correlated with the day 2 to 7 fold increase of antibody response for LPS IgA (r = 0.55, P = 0.01) and IgG (r = 0.46, P = 0.04), but not for LPS IgM, nor any CtxB antibody isotype responses (Figure 3C, D). We saw similar correlations between frequency of day 7 activated MAIT cells and increases in LPS IgA (Figure S2A; r = 0.50, P = 0.02) and IgG (r = 0.52, P = 0.02). On the other hand, we saw an inverse correlation between frequency of day 7 activated (CD38^+^) CD161^lo^Va7.2^+^ cells and LPS IgA (Figure S2B; r = −0.59, P = 0.006) and IgG (r = −0.47, P = 0.04) responses. As with MAIT cells, no correlations were seen for LPS IgM, nor any CtxB antibody isotype responses for either of the activated subpopulations.

## Discussion

Despite evidence demonstrating the importance of innate components on mediating the host response to cholera [14], [30]–[34], characterization of innate responses in cholera patients has been limited. MAIT cells are innate-like T cells that account for up to 10% of peripheral circulating T cells and are found in abundance in the human intestine [16], [35], [36]. Recent studies have identified microbially derived vitamin B metabolites as the MR1-restricted ligand presented to MAIT cells [37], [38]. Specifically, bacteria and yeast with a riboflavin biosynthetic pathway are able to activate MAIT cells, resulting in the rapid secretion by MAIT cells of cytokines including IFN-γ, TNF-α, and IL-17 [16], [18], with or without direct cytotoxic activity [26]. *Vibrio cholerae* O1, a causative agent of cholera, is a Gram negative bacterium known to have a riboflavin biosynthetic pathway [39].

In this study, we demonstrated that in both children and adults, the percentage of MAIT cells exhibiting CD38, an activation marker, are increased at day 7 after presentation with *V. cholerae* O1 infection compared to day 2 and healthy controls, and return to baseline levels by day 30. As cholera is a non-invasive disease, we hypothesize that the activation of MAIT cells is by *V. cholerae* riboflavin metabolites in intestinal tissues, and that the increase of activated MAIT cells in the peripheral blood at day 7 may reflect an increase in numbers of activated MAIT cells in the gut several days prior.

In observing the kinetics of MAIT cells in cholera patients, we showed that children exhibit a decrease in total circulating MAIT cells by day 7 after cholera that persists to at least day 90 following infection, whereas adults maintain a steady number of total MAIT cells in the blood during all time points sampled following cholera. This adult/child discrepancy was present even when MAIT cells were calculated as absolute counts per volume of blood. Our current study is, to our knowledge, the first description of MAIT cells in children with a mucosal infection, and the reason for the decrease of total MAIT cells in the peripheral blood of children with cholera, but not in adults with cholera, is uncertain. In studies using healthy controls as comparator groups, adults with pulmonary infections [18], [19], and bacterial sepsis [25], also have lower frequencies of circulating MAIT cells in the peripheral blood. Furthermore, adults with HIV infection exhibit a longstanding depletion of MAIT cells in the peripheral blood that persists despite antiretroviral treatment, while MAIT cell numbers in rectal mucosa are preserved [20], [21]. A previous cross-sectional study of circulating MAIT cells in healthy children suggests that the frequency of MAIT cells reach adults levels by 2 years of age [16], and in our study, we also found that healthy children (all age >2 years) have similar MAIT frequencies as healthy adults. Children in resource-limited areas are particularly prone to environmental (tropical) enteropathy [40], a disorder characterized by marked abnormalities in intestinal architecture and substance thought to be the result of repetitive insults and infections [41]. Whether recruitment of MAIT cells in peripheral blood to this intestinal environment in children with environmental enteropathy affects the MAIT cells in a manner distinct from that in adults is unclear. For instance, MAIT cells recruited to intestinal tissues undergo antigen-induced cell death upon bacterial stimulation *in vitro* [21]. Alternatively, it is also possible that recruitment of MAIT cells to the gut mucosa continues for a lengthy period after initial *V. cholerae* infection in children, resulting in the prolonged depletion from the circulation seen in child patients. In support of this, a recent report using a mouse model of *Francisella tularensis* infection showed that MAIT cells remained at high levels in the lungs even after clearance of infection [24].

Given recent studies demonstrating that CD3^+^CD161^lo^Vα7.2^+^ cells may be derived by MAIT cell co-culture with bacteria [20], that they have absent or reduced cytokine secretion compared with MAIT cells upon bacterial stimulation [20], [26], [27], and that they are associated with MAIT cell loss and functional exhaustion in HIV patients [20], we also evaluated the kinetics of this cell population in cholera patients and controls. We demonstrated that CD161^lo^Vα7.2^+^ cells are increased for up to 30 days following severe cholera before returning to baseline. Notably, we saw that fold changes of activated (CD38^+^) CD161^lo^Vα7.2^+^ T cells were inversely correlated with fold changes in MAIT cells. We also demonstrated that in both healthy child controls and children with cholera, activated CD161^lo^Vα7.2^+^ cells occur at higher frequency than in adults. However, as a recent study employing a MR1 tetramer demonstrated an absence of a CD3^+^CD161^lo^Vα7.2^+^ tetramer positive population in humans [37], suggesting their lack of MR1 restriction, details regarding the development and fate of these cells remain to be determined.

The role of the innate response on the formation of adaptive humoral responses in enteric infections is not well defined. A recent study in adults demonstrated that activation of MAIT cells is higher in *Shigella* vaccine responders compared to non-responders, when response was assessed using an LPS-specific IgA antibody secreting cell assay [26]. We investigated the association of MAIT cells with the magnitude of increase in antibody responses against two *V. cholerae* antigens: LPS, a T-independent polysaccharide antigen, and CtxB, a T-dependent protein antigen. We found that fold changes in circulating MAIT cells were correlated with the magnitude of increase in antibody response for anti-LPS IgA and IgG, but not IgM. Similar correlations were seen between frequencies of activated MAIT cells at day 7 and LPS IgA and IgG responses, and an inverse correlation was seen for activated CD161^lo^Vα7.2^+^ cells. Notably, we did not see any correlations between MAIT cells and LPS IgM, nor for CtxB antibody responses of any isotype. As such, MAIT cells may play a role in terminal class switching of antibody responses against polysaccharide antigens, although further study is required to clarify this association. It is also possible that MAIT cells may play a role in the T-independent differentiation of memory B cells, either directly or indirectly through interaction with antigen presenting cells. We also cannot rule out the possibility that B cells may play a role in the expansion of MAIT cells, given that activated B cells express the CD161 receptor ligand lectin-like transcript 1 [42], and MAIT cell expansion and accumulation in peripheral tissues has been shown to be B cell-dependent in animal models [36], [43].

There are several limitations to our study. First, our characterization of the kinetics of MAIT cells is limited to those in the peripheral circulation; we cannot comment on MAIT cell characteristics at the intestinal mucosa during cholera. Second, we did not exclude gamma-delta T cells in our phenotyping of MAIT cells, although the majority of these cells are not known to display Vα7.2^+^. Thirdly, we did not assess functionality of the cells, including the assessment of cytokine secretion and cytotoxicity markers following *ex vivo* and/or *in vitro* incubation of isolated MAIT cells with bacteria and *V. cholerae*-specific antigens; thus, comments regarding characteristics and markers of cellular exhaustion were based on previous reports. Fourthly, we quantified activation based on a single marker (CD38), though previous studies of MAIT cells have demonstrated that elevations in CD38 correspond to elevations of other activation markers such as HLA-DR and TIM-3 [20]. Fifthly, we did not perform serial blood draws in healthy controls; thus, the decrease in MAIT cell frequency seen in infected children could be due to factors other than cholera. Lastly, we did not have sufficient sample size to perform separate statistical analyses for adults and children with regard to the association between MAIT cell and antibody responses.

In conclusion, we found that following acute *V. cholerae* O1 infection, the frequency of MAIT cells in the peripheral circulation falls in children but not adults, and that in both children and adults, an increased proportion of circulating MAIT cells display an activation marker by day 7 following cholera. Importantly, we found a positive correlation of MAIT cell increase with the development of class-switched antibody responses to a T cell-independent antigen, but not to a T cell-dependent antigen, suggesting that MAIT cells may play a role in bridging innate and adaptive immune responses for T cell-independent antigens. Our results suggest that the mechanisms behind MAIT cell activation and exhaustion, the interaction of MAIT cells with effectors of adaptive responses, the potential role of MAIT cells at the mucosal surface during cholera, and the functional differences during cholera of MAIT cells among age groups all require further investigation.

## Supporting Information

Figure S1 Geometric mean of absolute counts of A) MAIT and B) CD4^−^CD161^lo^Va7.2^+^ cells of healthy controls and patients with severe cholera, separated by adults and children, expressed as cells per mm^3^ of blood, as i) all cells, and ii) activated (CD38^+^) cells. * P<0.05; ** P<0.01; *** P<0.001.
(TIF)

Figure S2 Class-switched antibody responses against LPS are correlated with activated MAIT cell responses. Correlation of fold changes in antibody response of cholera patients with A) day 7% activated MAIT cells and B) day 7% activated CD161^lo^Va7.2^+^ cells.
(TIF)
