# Supplementary figures and images for: Circulating Mucosal Associated Invariant T Cells Are Activated in Vibrio cholerae O1 Infection and Associated with Lipopolysaccharide Antibody Responses
Source: PLoS Negl Trop Dis. 2014 Aug 21;8(8):e3076. doi: 10.1371/journal.pntd.0003076 (PMC4140671; doi:10.1371/journal.pntd.0003076)

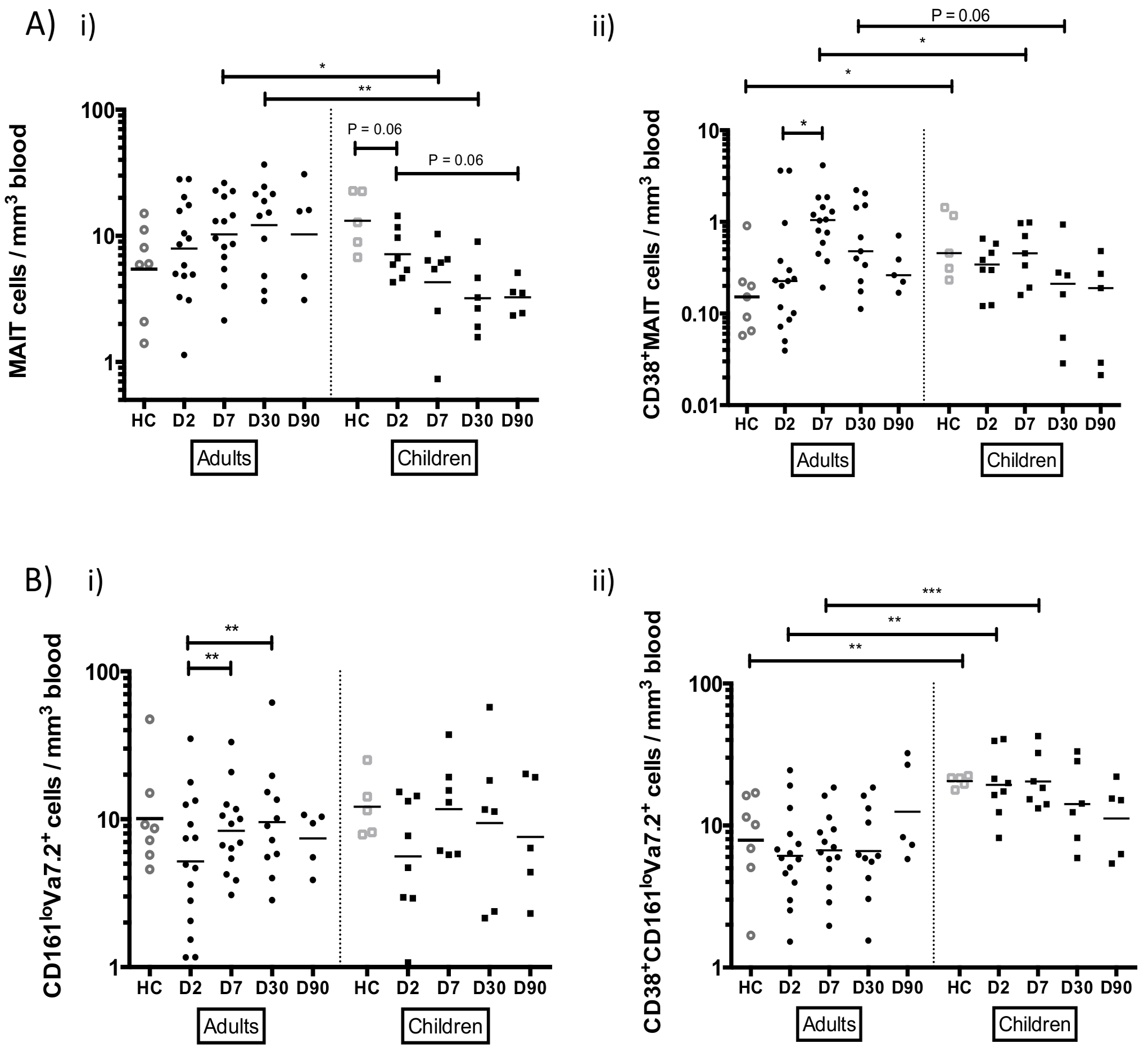

Supplement: Figure S1 — Geometric mean of absolute counts of A) MAIT and B) CD4−CD161loVa7.2+ cells of healthy controls and patients with severe cholera, separated by adults and children, expressed as cells per mm3 of blood, as i) all cells, and ii) activated (CD38+) cells. * P<0.05; ** P<0.01; *** P<0.001. (TIF) [file pntd.0003076.s001.tif]
